# Correlated evolution of nucleotide substitution rates and allelic variation in *Mhc-DRB *lineages of primates

**DOI:** 10.1186/1471-2148-9-73

**Published:** 2009-04-12

**Authors:** László Z Garamszegi, Natasja G de Groot, Ronald E Bontrop

**Affiliations:** 1 Department of Biology, University of Antwerp, Campus Drie Eiken Universiteitsplein 1, B-2610 Wilrijk, Belgium; 2 Department of Evolutionary Ecology, Estación Biológica de Doñana-CSIC, c/Americo Vespucio, s/n, 41092, Sevilla, Spain; 3 Department of Comparative Genetics and Refinement, Biomedical Primate Research Centre, PO Box 3306, 2280 GH Rijswijk, the Netherlands

## Abstract

**Background:**

The major histocompatibility complex (MHC) is a key model of genetic polymorphism. Selection pressure by pathogens or other microevolutionary forces may result in a high rate of non-synonymous substitutions at the codons specifying the contact residues of the antigen binding sites (ABS), and the maintenance of extreme MHC allelic variation at the population/species level. Therefore, selection forces favouring MHC variability for any reason should cause a correlated evolution between substitution rates and allelic polymorphism. To investigate this prediction, we characterised nucleotide substitution rates and allelic polymorphism (i.e. the number of alleles detected in relation to the number of animals screened) of several *Mhc *class II *DRB *lineages in 46 primate species, and tested for a correlation between them.

**Results:**

First, we demonstrate that species-specific and lineage-specific evolutionary constraints favour species- and lineage-dependent substitution rate at the codons specifying the ABS contact residues (i.e. certain species and lineages can be characterised by high substitution rate, while others have low rate). Second, we show that although the degree of the non-synonymous substitution rate at the ABS contact residues was systematically higher than the degree of the synonymous substitution rate, these estimates were strongly correlated when we controlled for species-specific and lineage-specific effects, and also for the fact that different studies relied on different sample size. Such relationships between substitution rates of different types could even be extended to the non-contact residues of the molecule. Third, we provide statistical evidence that increased substitution rate along a MHC gene may lead to allelic variation, as a high substitution rate can be observed in those lineages in which many alleles are maintained. Fourth, we show that the detected patterns were independent of phylogenetic constraints. When we used phylogenetic models that control for similarity between species, due to common descent, and focused on variations within a single lineage (DRB1*03), the positive relationship between different substitution rates and allelic polymorphisms was still robust. Finally, we found the same effects to emerge in the analyses that eliminated within-species variation in MHC traits by using strictly single population-level studies. However, in a set of contrasting analyses, in which we focused on the non-functional *DRB6 *locus, the correlation between substitution rates and allelic variation was not prevalent.

**Conclusion:**

Our results indicate that positive selection for the generation of allelic polymorphism acting on the functional part of the protein has consequences for the nucleotide substitution rate along the whole exon 2 sequence of the *Mhc-DRB *gene. Additionally, we proved that an increased substitution rate can promote allelic variation within lineages. Consequently, the evolution of different characteristics of genetic polymorphism is not independent.

## Background

The maintenance of genetic polymorphism is a challenging question for evolutionary biologists because its adaptive value is controversial [1]. The major histocompatibility complex (MHC) contains the most variable set of genes of known function in vertebrates, and, as such, it offers a unique opportunity to test competing evolutionary hypotheses of molecular adaptation [e.g. [2-4]]. As the system plays a crucial role in immune defence, the most important hypothesis of MHC polymorphism proposes that the selective pressure of parasites influences the population genetics of the MHC [2,5] at the levels of both substitution rate and allelic variation. This can act either via the selective advantage of heterozygous over homozygous individuals [6] or via negative frequency-dependent selection by which rare MHC alleles incur benefits against pathogen strains that evade common alleles [7]. Accumulating evidence from wild populations that show that non-synonymous substitution rates are higher than synonymous substitutions demonstrate that the MHC underwent balancing selection [3]. Moreover, field studies repeatedly report a link between the presence of particular alleles and individual fitness in terms of freedom from pathogens [8]. However, the number of alleles present in a population, may not be only influenced by parasite pressure, but may also be influenced by population size effects, gene flow, drift, bottleneck effects [2,3].

Independent of the selective pressures operating, nucleotide substitution rate and allelic variation are different phenomena, as the former defines the probability of nucleotide exchange in the DNA sequence, while the latter describes the number of alleles that are preserved functioning in the population or species. Hence, selection forces favouring MHC variability can affect substitution rates, which may have consequences for the accumulation of functioning alleles, but there may be various genetic mechanisms that generate new alleles. It remains difficult to elucidate the evolutionary causes and consequences of MHC polymorphism, mostly because the molecular mechanisms underlining its extreme allelic variation remain unknown. The functional part of the molecule that accomplishes peptide presentation (contact residues of the antigen-binding sites [ABS]) and displays most of the polymorphism is generally thought to be driven by pathogen-driven selective pressures [9]. The ABS contact residues exhibit a consistently higher rate of non-synonymous than synonymous substitutions, which is generally interpreted as evidence that mutations altering amino acid sequence are being positively selected, in contrast to silent mutations [8,10]. Although, the higher rate of non-synonymous over synonymous substitutions provides evidence that selection processes are operating that favour the establishment of polymorphism, such evidence does not demonstrate what the exact genetic mechanisms are. For example, it does not prove that different substitution rates at the ABS contact residues are particularly favoured by parasites or that high substitution rates directly promote allelic variation, e.g. through point mutations at the ABS contact residues. Substitution rates may also be resulted from gene flow or bottleneck effects, while the adaptive number of alleles and antigen-specificity should not be determined exclusively by alterations at the ABS contact residues. Changes in amino acid sequences in the protein binding groove may affect stereochemistry, and substitution rates within the groove but outside of the contact residues may have consequences for the three-dimensional positioning of the ABS contact residues [11]. Moreover, certain regions, such as the contact region with the T-cell receptor (TCR), may also display polymorphic residues. Hence, substitution at the TCR binding sites may affect the stability of MHC peptide-receptor complex and the subsequent intrathymic selection of the responding T-cell repertoire that ultimately determines antigen-specificity [12]. Importantly, not only single codons and mutations may be involved; it should also be considered that the exchange of longer stretches of nucleotide sequences via recombinations may generate allelic polymorphism [13,14].

Although selection in space and time of specific alleles (e.g. according to fluctuations in the parasite density) can in theory be manifested in the accumulation of many different alleles, it is not crucial to expect that selection pressures (due to parasite resistance for example) always enhance a tight evolutionary link between rates of nucleotide substitutions and allelic variation that is preserved at the population or species level. Accordingly, it does not necessarily mean that a high substitution rate can be translated into genetic polymorphism. If individuals with specific MHC allele or allele combinations enjoy selective advantages in a given environment, the fitness benefit accrued to a certain MHC genotype may not only lead to the accumulation of alleles but a certain allele (or allele combination) can also undergo directional selection and fixation [15-17]. Therefore, even if high substitution rate and the generation of new alleles is linked, it is not necessarily reflected by the maintenance of high allelic polymorphism that can be observed at the population level in terms of the number of alleles accumulated. Furthermore, the strength of selection on a single allele depends on the presence and net effect of other alleles within individuals [18]. The typical distribution of MHC alleles across individuals follows haplotype polymorphism (gene copy number variation), as due to past gene duplication events, individuals can possess more than two MHC alleles of the same lineage that are sometimes linked and that can only occur in specific combinations [19-22]. Hence, copy number variation and linkage can buffer or amplify the selective advantage/disadvantage of specific alleles (i.e. a polymorph haplotype has a higher chance of having a parasite-resistant allele than does an oligo- or monomorph haplotype). Consequently, an entire suite of evolutionary mechanisms that operate within the MHC genome may exist, which could shape observed levels of both substitution rates and allelic polymorphism.

For a better understanding of the evolution of MHC polymorphism, studies are needed that establish a link between substitution rates and allelic variation. Here, we accomplish this task and demonstrate a comprehensive analysis of exon 2 of the primate *DRB *gene. The DRB region exhibits the most elaborate polymorphisms in MHC class II genes, and these have been extensively characterised in primates, due to their importance in immunological research [23-31]. Based on an extensive survey of the literature, we counted the number of alleles relative to the number of animals sampled across 46 species, and sorted the corresponding nucleotide sequences into lineages (such as HLA-DRB1*03, Patr-DRB1*03 and Mamu-DRB1*03). Sequences that are derived from common ancestry in different species, that have known gene products and peptide-binding grooves that are highly similar, and that could therefore select the same peptide for T-cell activation, were considered to belong to the same lineage [32]. Based on the available sequences within each lineage, we characterised substitution rates at different sites in each species. We predicted that if elevated rates of non-synonymous substitutions at ABS contact residues generate allelic variation, there should be a positive relationship between these traits. We predicted a similar relationship for substitution rates at the non-contact residues, because substitutions or recombination events at these sites could be functionally important. We also predicted positive correlations between substitution rates at the ABS contact and non-contact residues, as selection exerted by pathogens can cause the joint evolution of nucleotide substitution and recombination events.

Statistically, we focused on variation *within *lineages, because comparisons *between *different heterologous or paralogous lineages may be difficult due to their incomparable function and physical position within the genome (e.g. we avoided comparing substitution rates between DRB1*03, DRB1*05 or DRB*W7). The main question was whether if for any reason a given lineage displays a high substitution rate at the ABS contact or non-contact residues, it also displays a high degree of allelic variation. Therefore, we performed our analyses at the lineage level, and used a General Linear Model (GLM) design to control for the fact that non-independent, multiple data were used from the same species and from the same lineage. This statistical setup also allowed us to assess the importance of species-specific and lineage-specific effects. Such effects, if detected, could reveal that a particular species maintains a more or less constant substitution rate across the possessed DRB lineages, or that a particular lineage displays similar substitution rates in those species in which it is present. The outcome of such a complex statistical design may be difficult to interpret, and is not completely independent of phylogenetic inertia due to the evolutionary relationships between species and lineages. Hence, we chose a single lineage (the best-characterised DRB1*03 lineage) to demonstrate the same relationships between substitution rates and allelic polymorphism in an interspecific context, when phylogenetic effects can be held constant. Additionally, we dealt with the fact that mixing data from different populations of the same species may yield systematically higher substitution rates and allelic diversity, because of population-specific selection factors that favour different MHC characteristics at each location [33]. To control for this unwanted within-species variation, we run additional analyses that used data from strictly single population-level studies. Finally, as a contrasting analysis, the predictions about relationships between different substitution rates and allelic variation by focusing on lineages of potentially non-functional loci (pseudogenes) were tested. If the corresponding nucleotide sequences are not translated into gene products and are thus not favoured by natural selection, one would expect that such nucleotide substitutions should be neutral. Therefore, it was predicted that in such non-functioning lineages, there would be no selection for high rate of non-synonymous substitutions that could result in correlations between different substitution rates and allelic variation. In the entire set of analyses, we controlled for differences in data quality by using statistical weights. This was necessary, as different studies screened different numbers of individuals raising heterogeneity in sampling effort.

## Results

### Patterns of substitution rates

**Figure 1:**
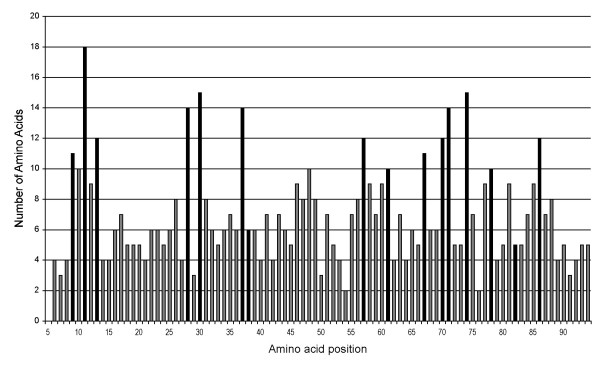
**Amino acid variability plot for 1174 MHC-DRB alleles from 46 primate species (all species and loci combined)**. The number of different amino acids found on the given position is shown. The considered contact residues of the antigen-binding sites (ABS) are drawn in black (see text for details).

The level of non-synonymous substitution rates along the ABS contact residues was on average 0.216 (s.e. = 0.012) substitution, range 0.000–0.807, N = 191 (data on 60 lineages across 43 species (for 3 species only one allele was available, thus we could not compute substitution rates). The level of synonymous substitution rates at the same sites was on average 0.072 (s.e. = 0.005) substitution, range 0.000–0.338, N = 191. Thus, the synonymous rate was on average 33.3% of the non-synonymous substitution rate. On the basis of sites that do not correspond to the ABS contact residues of the molecule, the patterns of distribution of substitution rates showed the following figures: non-synonymous, mean ± s.e. = 0.027 ± 0.001, range = 0.000–0.078; synonymous, mean ± s.e. = 0.026 ± 0.002, range = 0.000–0.115 (N = 191). The amino acid variability (i.e. the number of different amino acids found) of different sites of the molecule is shown in Figure 1.

### Species- and lineage-specific effects

**Figure 2:**
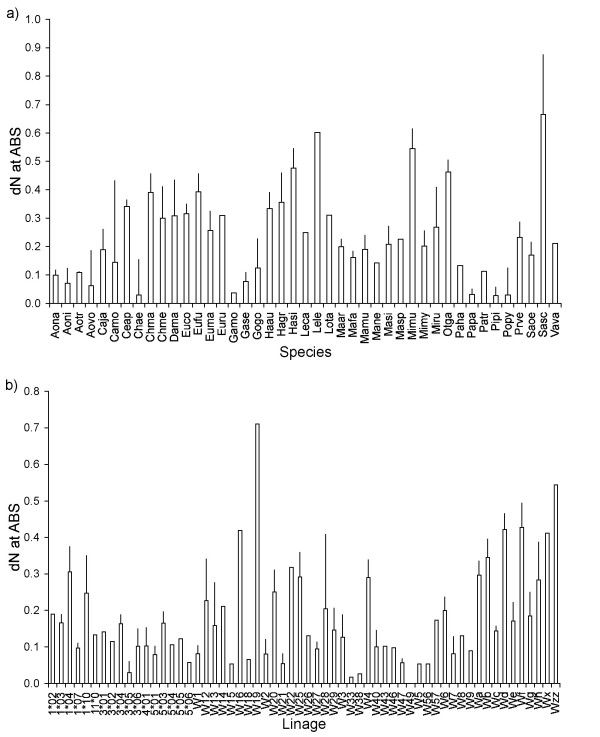
**Consistent within-species (a) and within-lineage (b) variations in non-synonymous nucleotide substitution rates at the contact residues of the antigen binding sites**. Columns are means, bars indicate standard errors. (a) Names are the four-letter abbreviations used for species names [27], and (b) lineage names.

We found that synonymous and non-synonymous substitution rates at different sites of the primate DRB molecule showed consistent within-species and within-lineage variations (Table 1, Figure 2). This indicates that there are species that systematically display higher substitution rates than others across a list of different lineages. However, there are lineages that can be typified by their high tempo of substitution in a range of species, while others are less variable. The species-specific patterns were robust because these were apparent when we only focused on HLA-orthologue lineages (Table 1). Nevertheless, the lineage-specific patterns may be caused by differences between the HLA orthologues and non-orthologous lineages (Workshop, 'W' lineages), since after the exclusion of the 'W' lineages, the systematic within-lineage variation appeared noticeably weakened (Table 1).

**Table 1:** Testing for consistent within-species and within-lineage variations in nucleotide substitution rates (N: non-synonymous, S: synonymous) of the MHC-DRB molecule at different regions (ABS: contact residues, non-ABS: non-contact residues)

All lineages	Species-specific effects	Lineage-specific effects
dN, ABS	F_42,148 _= 2.455, P < 0.001	F_59,131 _= 1.994, P < 0.001
dS, ABS	F_42,148 _= 1.628, P = 0.018	F_59,131 _= 1.544, P = 0.021
dN, non-ABS	F_42,148 _= 1.450, P = 0.055	F_59,131 _= 1.490, P = 0.031
dS, non-ABS	F_42,148 _= 1.893, P = 0.003	F_59,131 _= 2.568, P < 0.001
dN, all sites	F_42,148 _= 2.227, P < 0.001	F_59,131 _= 1.744, P = 0.005
dS, all sites	F_42,148 _= 1.503, P = 0.040	F_59,131 _= 1.782, P = 0.003

		

Only HLA othologous	Species-specific effects	Lineage-specific effects

dN, ABS	F_20,34 _= 2.260, P = 0.018	F_16,38 _= 0.798, P = 0.679
dS, ABS	F_20,34 _= 1.632, P = 0.102	F_16,38 _= 0.360, P = 0.984
dN, non-ABS	F_20,34 _= 2.195, P = 0.021	F_16,38 _= 1.412, P = 0.188
dS, non-ABS	F_20,34 _= 2.187, P = 0.022	F_16,38 _= 0.380, P = 0.980
dN, all sites	F_20,34 _= 2.765, P = 0.004	F_16,38 _= 0.783, P = 0.694
dS, all sites	F_20,34 _= 2.329, P = 0.015	F_16,38 _= 0.310, P = 0.993

Results form one-way ANOVA, when including all lineages and only human orthologues (i.e. after excluding W lineages).

The repertoire of lineages may vary in a species-specific manner, as primates closely related to humans are more likely to have orthologous *HLA-DRB *lineages, while prosimians basically have W lineages only. As a result, species-specific and lineage-specific effects may partially overlap, and thus species-specific effects may emerge via lineage-specific roles and vice versa. Therefore, we assessed the importance of these effects simultaneously in the same statistical model by using a GLM design, in which one factor can be controlled as a random effect. When we estimated species-specific effects independent of lineage-specific effects, we still found that variation at the species level explains a significant amount of variation in sequence polymorphism (Table 2). Similar patterns emerged in the reciprocal situation, when species effects were kept constant and the independent effects of lineages were estimated (Table 2). However, as we also found in the one-way ANOVA design, these latter effects were sensitive to the exclusion of W lineages.

**Table 2:** Testing simultaneously for consistent within-species and within-lineage variations in nucleotide substitution rates (N: non-synonymous, S: synonymous) of the MHC-DRB molecule at different regions (ABS: contact residues, non-ABS: non-contact residues).

All lineages	Species-specific effects	Lineage-specific effects
dN, ABS	F_42,91 _= 2.311, P < 0.001	F_59,89 _= 1.875, P = 0.004
dS, ABS	F_42,91 _= 1.809, P = 0.010	F_59,89 _= 1.572, P = 0.026
dN, non-ABS	F_42,91 _= 1.491, P = 0.058	F_59,89 _= 1.490, P = 0.044
dS, non-ABS	F_42,91 _= 1.690, P = 0.019	F_59,89 _= 2.628, P < 0.001
dN, all sites	F_42,91 _= 2.120, P = 0.002	F_59,89 _= 1.694, P = 0.012
dS, all sites	F_42,91 _= 1.908, P = 0.005	F_59,89 _= 2.135, P < 0.001

		

Only HLA orthologous	Species-specific effects	Lineage-specific effects

dN, ABS	F_20,18 _= 3.152, P = 0.009	F_16,18 _= 1.155, P = 0.381
dS, ABS	F_20,18 _= 1.632, P = 0.150	F_16,18 _= 0.356, P = 0.979
dN, non-ABS	F_20,18 _= 2.253, P = 0.044	F_16,18 _= 1.104, P = 0.417
dS, non-ABS	F_20,18 _= 3.058, P = 0.010	F_16,18 _= 0.878, P = 0.601
dN, all sites	F_20,18 _= 3.347, P = 0.006	F_16,18 _= 0.986, P = 0.507
dS, all sites	F_20,18 _= 2.842, P = 0.015	F_16,18 _= 0.645, P = 0.809

GLM results, when including all lineages and only human orthologues (i.e. after excluding W lineages)

These results imply that further analyses of substitution rates should consider species-specific and lineage-specific effects, which are applied in the following sections in addition to the control for heterogeneous sampling.

### Association between substitution rates at different sites

**Figure 3:**
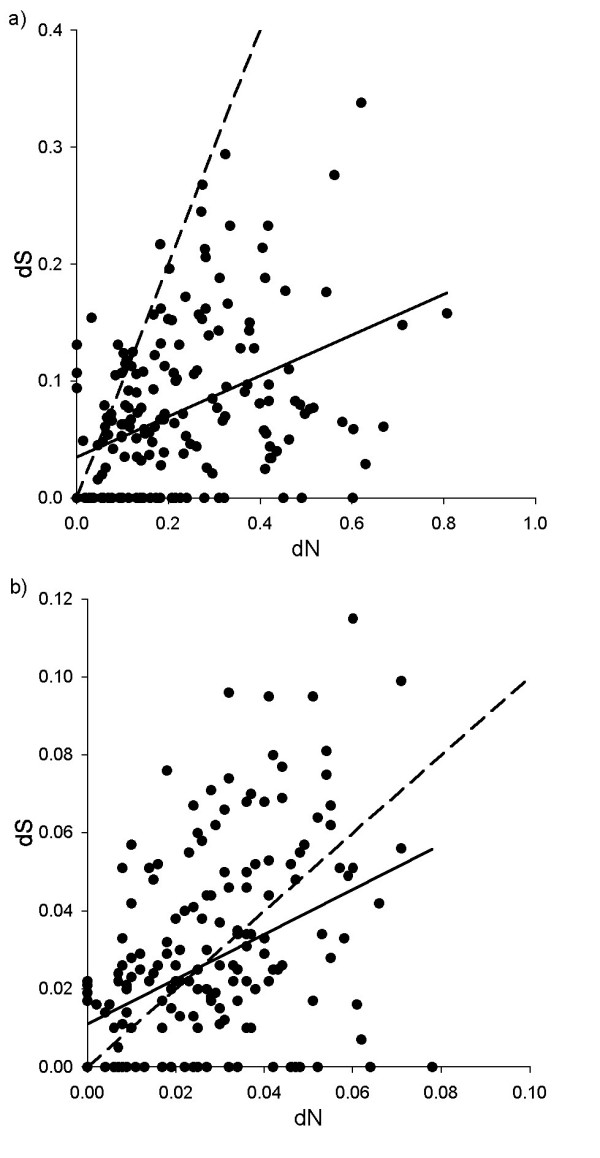
**The relationship between non-synonymous (dN) and synonymous (dS) substitution rates at the a) ABS contact residues and b) ABS non-contact residues**. Points are lineage-specific data when species are separated; solid lines are regression lines; dashed lines indicate y = x, which corresponds to neutrality. Regions below the neutral line denote positive selection pressure (dN > dS or dN:dS > 1).

As a strong indication for positive selection, the degree of non-synonymous substitution rates at the ABS contact residues was systematically higher than that of synonymous substitution rates at the same residues (paired-t_190 _= 13.19, P < 0.001). The mean of the dN:dS ratio was 5.728 (s.e. = 0.605). A positive correlation between dN and dS indicated that lineages that have a relatively large value for non-synonymous substitution rates also have a relatively large value for synonymous substitution rates (Table 3, Figure 3a). This may imply that selection favouring the accumulation of non-synonymous substitutions within a lineage also favours the accumulation of synonymous substitutions. However, the slope of the regression was significantly smaller than one (t_189 _= -18.25, P < 0.001), suggesting that a unit increase in dN is accompanied by an increase in dS that corresponds to a smaller degree of change (Figure 3a). Non-synonymous substitution rates at the ABS non-contact residues were also higher than synonymous substitution rates at the same residues, but these differences were less robust (paired-t_190 _= 2.82, P = 0.005; mean ± s.e. of the dN:dS ratio = 1.327 ± 0.101). We also observed a positive relationship between dN and dS (Table 3, Figure 3b), with the slope being smaller than could be expected from an isometric relationship between the two traits (t_189 _= -4.601, P < 0.001).

**Table 3:** Correlation between different estimates of nucleotide substitution rates (N: non-synonymous, S: synonymous) of the MHC-DRB exon 2 of the molecule at different regions (ABS: contact residues, non-ABS: non-contact residues).

All lineages, N = 191	dS, ABS	dN, non-ABS	dS, non-ABS
dN, ABS	0.630	0.815	0.619
dS, ABS		0.548	0.507
dN, non-ABS			0.656

			

Only HLA orthologues, N = 55	dS, ABS	dN, non-ABS	dS, non-ABS

dN, ABS	0.703	0.826	0.850
dS, ABS		0.635*	0.641*
dN, non-ABS			0.825

Effect sizes in the form of Pearson's correlation coefficient as derived from the corresponding GLM model, which included species and lineage as random effects and was weighted by sample size (log_10_-transformed number of individuals). All relationships are significant at the 0.001 < P level, except *, which corresponds to P < 0.01.

Interestingly, when variation between species and lineages was held constant and the analyses was weighted by sample size that balances differences in data quality between studies, the association between substitution rates was prevalent across the ABS contact and non-contact residues, and also in comparisons of substitutions of the same type (Table 3). Therefore, synonymous and non-synonymous substitution rates throughout the whole exon 2 sequence of the DRB molecule seem to covary with each other at the within-lineage level. Notably, these relationships were very similar when we focused on HLA-orthologues lineages (Table 3). The only difference observed was that synonymous substitution rates at the ABS non-contact residues were higher than non-synonymous substitution rates when W lineages were excluded (paired-t_54 _= -5.571, P < 0.001; mean ± s.e. of the dN:dS ratio = 0.658 ± 0.058). The same comparison for the ABS contact residues showed the opposite pattern when only human orthologues lineages were used (paired-t_54 _= 6.972, P < 0.001; mean ± s.e. of the dN:dS ratio = 4.176 ± 1.101).

### Substitution rates and allelic variation

**Figure 4:**
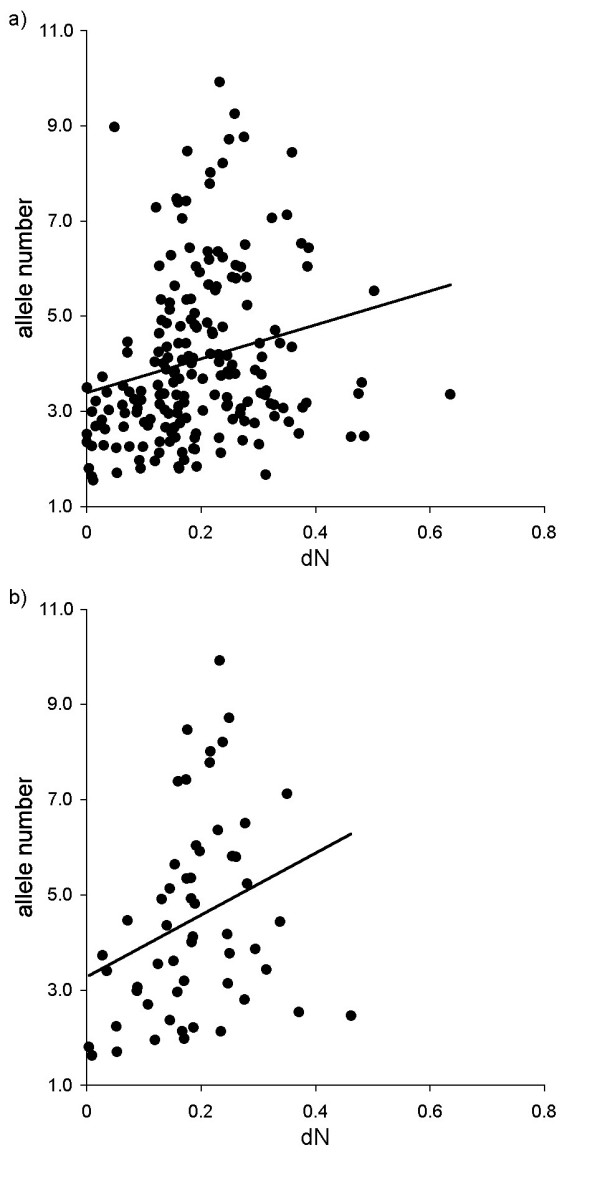
**The relationship between non-synonymous (dN) substitution rate at the contact residues of the ABS and allelic variation of DRB lineages when a) including and b) excluding non-human orthologous lineages (designated with 'W' workshop numbers)**. Allelic variation was estimated as the number of identified alleles relative to the number of animals sampled. Data were derived from the appropriate GLM model that included species-specific and lineage-specific effects and also the number of animals sampled (see text for details). Back-transformed data are shown; lines are regression lines.

If high rates of non-synonymous substitutions generate allelic variation within a given lineage, we predict a positive relationship between dN and the number of alleles relative to the number of animals sampled. Accordingly, we found that the degree of non-synonymous substitutions at the ABS contact residues was positively related to allelic variation estimated as a relative allele number, when we controlled for species-specific and lineage-specific effects and also for heterogeneous data quality by using statistical weights to correct for differences in sampling effort (r = 0.394, F_1,89 _= 16.380, P < 0.001, Figure 4a). This covariation at r ~0.3–0.4 was also prevalent when W lineages were excluded, but was not significant due to the much lower sample size reducing statistical power (r = 0.324, F_1,16 _= 1.878, P = 0.189, Figure 4b).

We also examined the possibility that the relationship between relative allele number (allelic variation) and substitution rate might be mediated by an artefact caused by the sensitivity of the estimation of dN to the number of alleles screened. We selected dN estimates that were derived from analyses of exactly five sequences. This threshold of five alleles was decided based on the balance between precision and sample size, as such samples may provide dN estimates of sufficiently high precision, and are available for a number of lineages that allow statistical analyses (N = 20). Hence, when the number of sequences is forced to be equal, it does not inflate the estimation of substitution rates. However, the corresponding sample size in terms of the number of animals screened for the detection of these five alleles showed considerable variation, and can be used to reflect allelic variation. If many animals have been analysed to detect these five alleles, it indicates that only a few alleles are present in a large population, which is equivalent to low allelic variation. In the opposite case, if five alleles are present in only a few individuals, one may assume that there are several alleles present in a larger sample, which implies that allelic variation is high. Therefore, when the number of alleles is held constant, the number of animals screened is an inverse estimate of allelic polymorphism. As such, this should correlate negatively with non-synonymous substitution rate if the latter affects allelic variation. Indeed we detected such a relationship (r = -0.492, N = 20, P = 0.028; when using statistical weights, r = -0.466, N = 20, P = 0.038). Due to the low sample size available, we were unable to deal with variation between species and lineages. However, this correlation may show that the relationship between relative allele numbers and substitution rates is unlikely to have been caused by the number of alleles analysed.

Given the high degree of association across different substitution rates and across different sites (Table 3), we predicted finding associations between allelic variation in terms of relative allele number and different substitution rates. These predictions were supported statistically (dS at contact residues of the ABS: r = 0.438, F_1,89 _= 21.127, P < 0.001; dN at non-contact residues: r = 0.410, F_1,89 _= 17.967, P < 0.001; dS at non-contact residues: r = 0.415, F_1,89 _= 18.533, P < 0.001, species and lineage effects were held constant and the analyses were weighted by sample size). When W lineages were excluded comparable effect sizes emerged, but were non-significant probably due to the resulting low sample size (dS at contact residues of the ABS: r = 0.318, F_1,16 _= 1.799, P = 0.199; dN at non-contact residues: r = 0.480, F_1,16 _= 4.800, P = 0.043; dS at non-contact residues: r = 0.307, F_1,16 _= 1.667, P = 0.215). Therefore, the exclusion of W lineages raised issues about statistical power, but the magnitude of the relationships of interest remained unaffected.

### Removing phylogenetic effects: comparative analyses of DRB1*03 polymorphism across species

Although employing random factors in the GLM models can eliminate the problem of the use of multiple entries from the same species and lineages that would cause pseudoreplication, species (and also lineages) may share a phylogenetic history that can still render data points to be statistically non-independent of each other. To handle this potentially confounding effect of phylogenetic inertia, we repeated the above correlations at the interspecific level by controlling for the phylogenetic associations between species and by using the DRB1*03 lineage. Therefore, the common descent of species can be eliminated by modern comparative methods, while a strict focus on a single lineage does not require considering problems that are due to the relationship between lineages.

To perform interspecific analyses, we therefore selected the best-characterised lineage and tested the same predictions about the evolution of MHC-DRB lineages across species. At least two alleles within the DRB1*03 lineage were available in 18 species, for which we had information on nucleotide substitution rates (dN at contact residues of the ABS, mean ± s.e. = 0.166 ± 0.023, range = 0.000 ± 0.306; dS at ABS contact residues, mean ± s.e. = 0.026 ± 0.004, range = 0.000–0.055; dN at ABS non-contact residues, mean ± s.e. = 0.064 ± 0.013, range = 0.000 ± 0.162; dS at ABS non-contact residues, mean ± s.e. = 0.041 ± 0.006, range = 0.000–0.077). As expected, the dN at the ABS contact residues was consistently higher than dS (paired-t_17 _= 4.779, P < 0.001), leading to the mean of dN:dS ratio of 3.704 (s.e. = 0.745). However, when focusing on the non-contact residues, we found the reverse (paired-t_17 _= -3.080, P = 0.007, mean ± s.e. dN:dS ratio = 0.679 ± 0.094).

**Figure 5:**
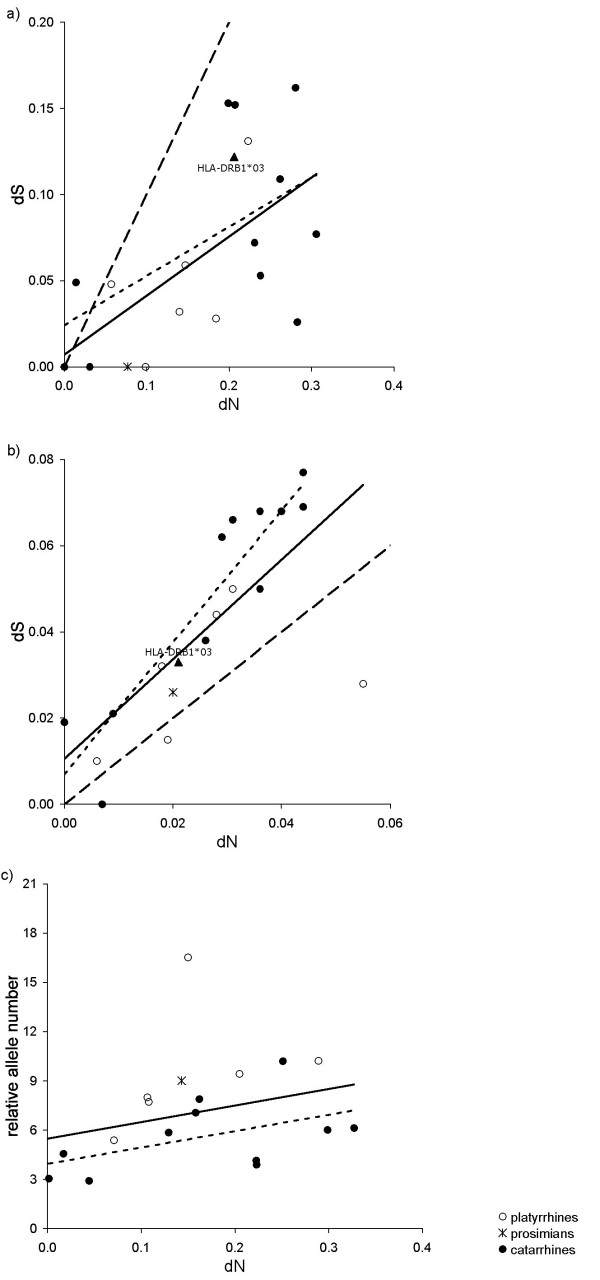
Natural selection and correlated evolution of substitution rates and allelic variation in the DRB1*03 lineage of primates. The interspecific relationship between non-synonymous (dN) and synonymous (dS) substitution rates at the a) ABS contact residues and b) ABS non-contact residues. c) The association between non-synonymous (dN) substitution rate at the ABS and allelic variation of the lineage (the number of alleles when the number of animals were held constant). Each data point represents the polymorphism of the DRB1*03 lineage in different species, and was obtained from the appropriate statistical model (see text) that considers the phylogenetic relatedness of species. Symbols represent different taxonomical groups of primates. Solid line is the regression line laid on the entire dataset; dotted line is the regression line that is obtained for New World primates only; dashed line indicates y = x. For illustration; we also included data from human (HLA-DRB1*03, HLA-DRB1*11, HLA-DRB1*12, HLA-DRB1*13 and HLA-DRB1*14 combined; data were subtracted from the IMGT/HLA database, ). We could not add human data to c), because the number of individuals screened for the 324 detected alleles is unknown and thus relative allele numbers cannot be calculated.

Similar to our earlier findings, estimates of substitution rates were strongly intercorrelated, when the phylogenetic relatedness and the heterogeneous sampling of species were controlled in an appropriate PGLS model (Table 4, Figure 5). Moreover, we could confirm that the slope of the dN-dS regression line corresponding to the ABS contact residues was smaller than unity (raw species data: t_16 _= -4.444, P < 0.001; phylogenetic control: t_16 _= -2.845, P = 0.012). In contrast, this was not the case for the regression based on non-contact residues, which appears to be isometric (raw species data: t_16 _= -0.586, P = 0.566; phylogenetic control: t_16 _= 0.587, P = 0.566).

**Table 4:** Correlation between different estimates of nucleotide substitution rates (N: non-synonymous, S: synonymous) of the DRB1*03 lineage when sampled across all available species (N = 18) and when only catarrhines are considered (N = 11).

All species, N = 18	dS, ABS	dN, non-ABS	dS, non-ABS
dN, ABS	0.561*	0.927***	0.771***
dS, ABS		0.480*	0.418†
dN, non-ABS			0.749***

			

Only catarrhine species, N = 11	dS, ABS	dN, non-ABS	dS, non-ABS

dN, ABS	0.673*	0.947***	0.800**
dS, ABS		0.689*	0.679*
dN, non-ABS			0.778**

ABS: contact residues, non-ABS: non-contact residues. Effect sizes as estimated from the PGLS model that adjusted for the phylogenetic relationships of species and used statistical weights (log_10_-sample size) in a combination that offered the best fitted to the data based on maximised log-likelihood. ***: P < 0.001; **: P < 0.01; *: P < 0.05; † < 0.1.

We tested for the relationship between substitution rates and allelic variation. Again, we used the number of alleles relative to the number of animals sampled as an estimate of allelic polymorphism, and found that it was positively related to dN at the ABS contact residues (PGLS model combining phylogenetic control and statistical weights: partial r = 0.610, N = 18, P = 0.009, Figure 5). This pattern was also seen in relation to the other substitution rates (PGLS models combining phylogenetic control and statistical weights: dS at contact residues of the ABS, partial r = 0.748, N = 18, P < 0.001; dN at non-contact residues, partial r = 0.614, N = 18, P = 0.009; dS at non-contact residues, partial r = 0.773, N = 18, P < 0.001).

It has been claimed that only alleles of the DRB1*03 lineage from humans, apes and Old World monkeys are orthologous, and in New World monkeys and lesser primates all DRB1*03 alleles are generated by convergent evolution [29,34]. However, when we applied a more narrow sense criterion for orthology and restricted our analyses to lineages from catarrhine species only, the detected patterns were very similar in terms of the magnitude of the effect detected (see Table 4 for the correlations between substitution rates; the relationship between dN at the at contact residues of the ABS and relative allele numbers, PGLS model: r = 0.573, N = 11, P = 0.069, Figure 5). In general, a strict focus on the DRB1*03 lineage in combination with statistical control for phylogenetic relationships and heterogeneous sampling of species provides very similar results as to the DRB-wide analyses (Figure 3) that factored out variations due to species-specific and lineage-specific effects.

### Removing effects due to within-species variation: analyses at the population level

In previous approaches, we included data that originated from different populations. However, sampling more than one population per species may result in the detection of more alleles and higher substitution rates, simply because different populations are adapted to different environments with different MHC characteristics. To remove this confounding effect of within-species variability, we restricted our analyses to clear population level studies. However, using such a restriction caused a considerable amount of information loss and a reduction of statistical power, because only a few studies were available for this purpose (see Methods).

We found 10 different lineages across 4 species that could be divided into well-characterised populations, and resulted in N = 17 independent data points (i.e. population-specific combination of lineages). We calculated nucleotide substitution rates that were similar to those we obtained in the above analyses (dN at contact residues of the ABS, mean ± s.e. = 0.265 ± 0.056, range = 0.000 ± 0.674; dS at ABS contact residues, mean ± s.e. = 0.118 ± 0.022, range = 0.000–0.253; dN at ABS non-contact residues, mean ± s.e. = 0.026 ± 0.006, range = 0.000 ± 0.081; dS at ABS non-contact residues, mean ± s.e. = 0.042 ± 0.009, range = 0.000–0.106). Again, the dN at the ABS contact residues was consistently higher than dS (paired-t_16 _= 3.214, P < 0.005, mean ± s.e. dN:dS ratio = 3.220 ± 0.655), and the analogue comparison at the non-contact residues revealed the opposite tendency (paired-t_13 _= -2.640, P = 0.018, mean ± s.e. dN:dS ratio = 0.561 ± 0.101). We could also replicate the generally positive relationship between different estimates of substitution rates, when species-specific effects were held constant and when we controlled for differences in sample size by using statistical weights (ABS contact residues: r = 0.739, P = 0.003, N = 17, regression slope is smaller than unity, t_15 _= -3.560, P = 0.003; non-contact residues: r = 0.748, P = 0.002; regression slope is isometric, t_15 _= 0.121, P = 0.905). Finally, we also found a positive association between the number of alleles relative to the animals sampled and dN at the ABS contact residues (r = 0.771, N = 17, P = 0.002, when species-specific effects and heterogeneity in sample size were controlled). These results remained qualitatively unchanged, when we excluded data from gorilla populations, in which one dominant male may father the majority of offspring that may be a confounding factor (correlations between different substitution rates: r = 0.768 – 0.903, P = 0.004 – 0.0001, N = 12; correlation between dN at the ABS contact residues and allelic variation: (r = 0.781, N = 12, P = 0.013).

### Contrast analysis with the *DRB6 *locus

The above series of analyses was repeated by using potentially non-functional nucleotide sequences. We found 4 different *DRB6 *lineages with at least two alleles (from DRB6*01 to 04) across 10 species providing us with a sample size of 14 species-specific combinations of lineages. Based on the presence of several shared characteristics [30], we assumed that these sequences are non-functional. Confirming this assumption, the calculated nucleotide substitution rates at the contact residues of the ABS were lower than in the case of functional lineages (dN at contact residues of the ABS, mean ± s.e. = 0.028 ± 0.008, range = 0.000 ± 0.098; dS at ABS contact residues, mean ± s.e. = 0.068 ± 0.037, range = 0.000–0.441; dN at ABS non-contact residues, mean ± s.e. = 0.034 ± 0.010, range = 0.000 ± 0.101; dS at ABS non-contact residues, mean ± s.e. = 0.049 ± 0.015, range = 0.000–0.168). Moreover, the dN at the ABS contact and non-contact residues was not consistently higher or lower than dS at the same residues (contact residues of the ABS, paired-t_13 _= -0.226, P = 0.825; non-contact residues, paired-t_13 _= -1.100, P = 0.291). Contrary to previous results with potentially functioning *DRB *lineages, there was no indication for a generally strong positive relationship between different estimates of substitution rates at the ABS contact residues, when lineage-specific effects were held constant and when we controlled for differences in sample size by using statistical weights (ABS contact residues: r = 0.194, P = 0.510, N = 14; non-contact residues: r = 0.815, P < 0.001; there was not enough variation in the data to control for species-specific effects). Finally, we found no association between the relative number of alleles and dN at the ABS contact residues (r = -0.035, N = 14, P = 0.921, when lineage-specific effects and heterogeneity in sample size were controlled).

## Discussion

Relying on the information currently available in the literature and the annotated sequences in the NHP-MHC and GenBank databases, we processed a comprehensive dataset on MHC-DRB allelic polymorphism and nucleotide substitution rate in primates. We collected MHC data from 51 scientific studies representing exactly 2500 animals and 1174 sequences. Our aim was to determine the degree of correlation between different estimates of substitution rate and allelic polymorphism within lineages. We found that substitution rates along exon 2 of the MHC-DRB gene vary consistently both within species and lineages. The same species appear to display similar degrees of substitution across different lineages, while the same lineage also possesses similar nucleotide variations in different species. Therefore, we adopted a GLM design, in which we could control for such species-specific and lineage-specific effects, and thus could focus statistically on variations between alleles, which drive polymorphism within a lineage. In addition, by using statistical weights in the analyses, the confounding effect of heterogeneous data quality arising from that different numbers of animals were screened for MHC alleles in different species was removed. These controlled analyses revealed that non-synonymous substitution rates at sites that correspond to the contact residues of the antigen-binding pocket of the translated protein were consistently higher than synonymous substitution rates at the same sites. The existence of such patterns was not so obvious at ABS non-contact residues, as dN:dS ratio varied across analyses. However, it is remarkable that in all tests a strong relationship between substitution rates of different types and at different sites was observed. Furthermore, different estimates of substitution rates also predicted allelic variation. Additionally, we demonstrated that such relationships were present, when we focused on a single lineage (DRB1*03) and simultaneously controlled for phylogenetic relationships of species and heterogeneous sampling by modern comparative approaches. These patterns also emerged when we restricted our analyses to population-specific data. Contrary, these correlations were absent in a series of contrasting analyses, in which we focused on the well-characterized lineages of the *DRB6 *pseudogene. Our findings indicate that any selection factor that has consequences for the substitution rate at a given site may also have consequences for the substitution rate at another site and also for the generation of new alleles within a functional lineage. Therefore, this is the first study that shows that different traits of genetic polymorphism evolve together, which has important implications for the evolutionary ecology of the MHC.

Extensive sequence variation at sites where antigen recognition occurs is a typical feature of the MHC [8]. This indicates that balancing selection operates on this region of the genome, and that the recognition of pathogens may have played a key role in the evolution of MHC polymorphism. At the ABS contact residues, almost all primate lineages with available data exhibited a dN:dS ratio greater than one (Figure 3a), with at least a threefold difference on average between non-synonymous and synonymous substitution rates. This should be considered robust when compared to other genes, such as reproductive or metabolic genes [1]. Sequence polymorphism at the ABS contact residues have been suggested to persist over a long period, and some evolutionary constraints may have favoured each species at a certain period to maintain a set of alleles that allow diverse amino acid composition [3]. Interestingly, selection pressures that favour a high degree of substitution rates to alter the amino acid sequence of the functionally important part of the molecule also favour substitution rates of different types at the ABS non-contact residues. This joint evolutionary response in terms of the accumulation of substitutions may be a genetic mechanism that ultimately generates new alleles.

Although sequence variation at the ABS non-contact residues was less emphasised than at the contact residues, it showed substantial correlated variation. Hence, selection factors affecting sites outside the contact residues should also accompany selection factors that generate MHC allelic variation. Therefore, increased rates of non-synonymous substitutions at the ABS contact residues are not the only mechanisms that result in variable sequences or/and more alleles. First, non-synonymous changes in non-contact residues may alter the contact region with the T-cell receptor, with consequences for antigen specificity [12]. The MHC class II molecule *per se *shows, as compared to the MHC class I molecule, limited specificity and is known to be more promiscuous [35,36]. The perfect discrimination of antigens is accomplished during the maturation of the immune system, when the peptide-MHC complex is recognised by highly specific T cells. Indeed, even a single change in the MHC sequences that influence receptor binding may be deleterious for recognition [37]. Second, the results showed that not only the effect of non-synonymous substitutions but that of synonymous changes should also be considered, as such substitutions occur parallel to increasing allelic variation. Variation in synonymous rates may be driven by differences in generation time or variable DNA damage due to distinct metabolic rates [38,39]. As such, for an adaptive allelic variation to be maintained even at a constant non-synonymous substitution rate, species may need to decrease generation time or metabolic rate or to introduce effective error-correcting machinery. Third, the accumulation of synonymous substitutions escorting the accumulation of non-synonymous substitutions may also be caused by genetic hitchhiking-like effects, whereby polymorphism at selectively natural sites tightly linked to sites experiencing strong selection may be affected [40]. Fourth, changes in dN and dS along the molecule do not exclusively cover alterations at single codons, but they can reflect the role of intragenomic recombinations, which may involve longer nucleotide sequences [41]. Particularly high levels of recombination may exist in MHC genes, which can be an important means of producing high allelic variation [13].

In any case, the possibility that the correlation between synonymous and non-synonymous substitutions occurs because they diverged and accumulated over the same period can be excluded. The slope of the corresponding regression line was systematically different from unity (Figures 3 and 5), which suggests that one substitution trait increased considerably less than the other. This is in contrast to time effects that would have raised symmetrical selection pressures on different substitution rates and would have resulted in isometric correlations. Moreover, our analyses focusing on the interspecific variation of DRB1*03 variability showed that the relationships were present when we controlled for the phylogeny of species. This phylogenetic control also applies statistical control for the divergence time of species, as the used phylogenetic tree relied on estimated divergence times [42].

We demonstrated that measures of sequence polymorphism vary in a species-specific manner. Other studies have observed remarkable patterns of interspecific variation in MHC variability, and have suggested that these differences must be interpreted in the context of life history, demography and ecological niches of species [13,15]. In particular, pathogens have been long suspected to be responsible for maintaining allelic variation at the MHC [6]. For example, nematode burden is connected with distribution of *Mhc-DRB *alleles in several taxa [43-48] including primates [49]. Heavily parasitised species maintain more MHC alleles to function against pathogens, which can be a result of altering host-parasite cycles that favour the preservation of different alleles at varying frequency [7]. Therefore, species-specific pressure due to disease risk may be a likely candidate that should select for allelic polymorphism and that should have consequences for nucleotide substitution rates that maintain such polymorphism. However, parasitism is not the only factor that necessitates the maintenance of allelic variation. Another set of fundamental hypotheses of allelic polymorhism concerns the importance of mate choice for particular genotypes or gene combinations that enhance parasite resistance or outbreeding [4]. As a result, selection for increased reproductive success via advantageous MHC traits should not exclusively favour the functional (i.e. ABS) sites of the molecule but all genetic traits that lead to more alleles. As such, inbreeding avoidance can promote increased substitution rates at other parts of the molecule. However, the key evolutionary factors that shape interspecific variation in substitution rate and allelic polymorphism remain to be identified. Phylogenetic comparative approaches [e.g. [50]] may offer a powerful tool to relate components of life history, sexual selection and parasitism to species-specific estimates of MHC polymorphism at different levels. Our results showing consistent within-species variation may provide a starting point for such studies, as they prove that species-specific estimates make biological sense.

We also found some evidence that selection pressures may act differently on distinct lineages. Most importantly, the strongest diversification appears between lineages that are orthologous and that are not orthologous with the HLA lineages. We know little about the status of MHC sequences with workshop numbers, as they may involve loci/lineages for which no obvious equivalents are known in the HLA system. Therefore, the lineage-specific patterns may be explained by the fact that different lineages represent various loci with dissimilar functions [see [51]]. In any case, we statistically controlled for any noise that could arise from the between-lineage variation.

Although, we processed an enormous amount of information on MHC sequence polymorphism that is currently available in the primate literature and considered several confounding factors at the analytical levels, some limitations that currently cannot be treated quantitatively warrant attention. For example, it may be that some of the primate species included in this study have experienced severe bottlenecks and, as a consequence, current levels of MHC diversity may not be the result of pathogenic selective pressures but may instead be the result of other microevolutionary forces. However, our study was not designed to specifically test the effect of parasites, but to identify correlations between MHC traits whatever the selective factor is in the background. Further investigations are needed to demonstrate how selection forces due to parasites and/or other factors can contribute to the correlated evolution of substitution rate and allelic variation at the MHC. Moreover, recently, it became evident that the *DRB *genes in primates may represent relative young entities, and must have arisen from a complex series of duplications [52,53]. As a consequence, some primates may appear to have two alleles of an apparently similar locus on one chromosome. However, the exon 2 sequences of these genes represent old entities predating primate speciation, and during primate evolution, they are continuously sprinkled over the duplicated genes by recombination-like processes [52]. The identification of gene duplications and the degree of haplotype polymorphism requires the screening and genotyping a large number of animals, a criterion with which only few model species meet. Detailed haplotype studies in rhesus macaques recorded only limited evidence for haplotypes harbouring *DRB *genes with exon 2 sequences grouping into the same lineages [54,55]. As such the performed analysis with exon 2 sequences of the *DRB *genes seems to be a legitimate approach. If more information have been gathered on the distribution of expressed alleles across individuals at the population level, the appropriate control for gene duplication will be possible.

## Conclusion

In conclusion, we have shown that nucleotide substitution rates at exon 2 of the *Mhc-DRB *gene can be dependent on the species and the lineage considered. Despite this, there was a clear indication that MHC allelic variation and nucleotide substitution rates follow parallel evolutionary routes. Allelic variation can be achieved by mechanisms that involve regions with both ABS contact and non-contact residues, and affect both synonymous and non-synonymous substitution rates. Hence, if a *DRB *lineage is selected for any reason (e.g. due to parasite pressure), and such selection factors shape nucleotide substitution of any kind, it will have consequences for substitution rates at different sites, as well as on the number of alleles generated within the lineage on an evolutionary time scale and maintained at the population level. The comprehensive analysis of MHC polymorphism of primate lineages generally supports theories that concern the concerted evolution of genetic traits. Further analyses are needed to identify the selection factors that are responsible for the correlated evolution of MHC traits, and to correct for potentially confounding factor of gene duplication.

## Methods

### MHC Data

We extracted information on MHC-DRB for prosimian, catarrhine and platyrrhine primates from the literature up to 31 May 2007, in an attempt to recover all published data. We relied primarily on references listed in the IPD/MHC database [, [56]], which we extended with additional sources as identified through systematic literature searches in Web of Science and GenBank. From each paper, we gathered information on the number and origin of animals sampled and the number of alleles (e.g. DRB1*0301, DRB3*0504, DRB*W706, or DRB*Wb01) detected in each lineage (e.g. DRB1*03 within the *DRB1 *locus, DRB3*05 within *DRB3 *locus, DRB*W28 and DRB*W7 or DRB*Wa and DRB*Wb within DRB*W). Sequences with a 'W' (workshop number) represent alleles or loci for which no human (HLA) orthologues exist [27]. However, we included them in our analysis because they do appear to form lineages [24,29,31,57,58], and thus our working hypothesis and prediction about the relationship between sequence and allelic polymorphism at the within-lineage level is applicable. Moreover, our statistical approach allowed a control for differences between HLA orthologues and non-orthologues lineages. We also did the analyses after excluding W lineages.

For a standard data collection and allele categorisation, we followed the universal MHC nomenclature [27,59] and the most recent taxonomy [[42], we added *Microcebus myoxinus *as separate species as suggested by [60]]. If the nomenclature in the source paper deviated from the standard, we adopted allele names as adjusted by the IPD/MHC, or categorised alleles based on sequence similarity with known alleles of closely related species. Recent taxonomy does not always fit with what had been used in the sources (e.g. the new taxonomy considers a single *Papio *species [42], while *P. anubis, P. cynocephalus, P. hamadryas *and *P. papio *were earlier treated as different species [57]). In such cases, we recategorised alleles according to the new scenario, and within a species we only counted alleles that had a non-identical nucleotide sequence. As the origin of lineages is occasionally unclear, it is difficult to identify the orthologous sequences across species. Testing our hypothesis, however, does not require orthologous lineages, as it corresponds to the general question of whether a selection pressure selects for increased sequence variability within a given lineage and also favours allelic polymorphism within the same lineage (i.e. we were not interested in identifying the lineage under selection). We assumed that diverse selective mechanisms would operate in different species and in different lineages, because the combination of species-specific and lineage-specific mechanisms that should be expected to result in that specific MHC structure appears to be favoured as polymorphs in each species (e.g. *DRB1 *lineages in Hominoids; DRB*W sequences in Lemurids and New World monkeys). Moreover, multiple data from the same species or lineage are non-independent, and so their use in statistical analyses raises issues about pseudoreplication [61]. Hence, we applied statistical approaches that allow control for the variation between species and between lineages (see below), with the result that the tests would permit implications independent of particular species or MHC lineage. The advantage of working with lineages is that variation in gene copy number is low, since heterozygote individuals can only possess a maximum of two alleles per lineage [e.g. [57,62-65]]. To deal with the non-independence of data points due to common descent, we used comparative analyses to remove phylogenetic effects (see below).

For each allele, we obtained the corresponding exon 2 nucleotide sequences from GenBank, and aligned them with MEGA version 3 [66] by following the source paper and IPD/MHC database. The alignments and the corresponding references used in this study are available as supporting material (Additional files 1 and 2). Each sequence was first checked for codon or nucleotide insertions and deletions and for premature stop codons. Alleles with these alterations are known as pseudogenes, and may code non-functional proteins [67]. Therefore, these alleles together with those from the DRB6 locus were excluded from the analyses of functioning sequences. However, DRB6 alleles were used in the contrast analyses of non-functioning lineages (see below). We used the Nei and Gojobori [68] method with the Jukes and Cantor [69] correction to calculate the rate of non-synonymous (dN) and synonymous (dS) substitutions as well as their ratio (dN:dS). We calculated these estimates of substitution rate and positive selection separately for the ABS contact and non-contact residues. We considered the following 16 ABS contact residues to be relevant: 9, 11, 13, 28, 30, 37, 38, 57, 61, 67, 70, 71, 74, 78, 82, 86 [70,71], while the remaining codons were treated as non-contact residues. These sites were labelled in the mode of "Select genes and domains" in MEGA. Some studies have suggested that other sites may be considered as contact residues in certain species, due to their high amino acid polymorphism [e.g. [62,72-74]]. However, functional and stereochemical evidence for these alternative sites is lacking, and their general applicability is limited. In the majority of species, we achieved the highest dN when we used the traditional sites, which generally appear to be the most polymorphic (difference in the number of amino acids detected between the considered contact and non-contact sites: t_88 _= 9.76, P < 0.001; [Figure 1]).

### Statistical and comparative analyses

Prior to the statistical analyses, all variables were appropriately transformed (i.e. we applied square-root-transformation for substitution rates, and log_10 _transformation for the number of alleles and animals sampled). We created a dataset at the level of lineage by entering the corresponding allele counts, substitution rates and number of animals for each lineage screened. We used a one-way ANOVA to identify species-specific and lineage-specific effects separately, in which we tested for consistent variation in substitution rates within species or lineages as categories. We built a General Linear Model (GLM) to deal with species and lineage effects simultaneously, and used them as random factors to control for their unwanted effects [75,76]. As we were interested in the relationships between different substitution rates, we entered the focal trait as the dependent variable, while the other was used as independent variable (main factor) together with species and lineage categories (random factors). Data quality varies from study to study, as the number of animals screened ranges from few individuals to several hundreds, which may affect the reliability of the calculated substitution rates from these samples. However, statistical approaches are available that allow control for heterogeneous data quality, as data points in the analyses can be weighted based on the corresponding sample size [75,77]. Therefore, we also included the log_10_-transformed number of animals sampled in the GLM model as statistical weight (we used log-transformed data to avoid overemphasizing data points with very large sample size). From this model, we derived the statistics for the relationship in question, and translated it into a Pearson's correlation coefficient to illustrate the degree of association between traits in a form of standard effect sizes [sensu [78]]. When we assessed the link between substitution rates and allelic variation, we created a model with the number of alleles as the dependent variable, and with the investigated substitution rate as the independent variable. The model also included the number of animals, because the absolute number of alleles found in a species is clearly dependent on the number of animals sampled (F_1,44 _= 45.29, P < 0.001). Hence, we removed such sample-size effects, and by entering the number of animals, we could estimate the relative (residual) number of alleles as a measure of MHC allelic variation. As earlier, the model also incorporated the two random factors to account for variations between species and lineages and used weights to balance sample size differences. We report partial correlations for the focal traits, which should reflect the relationship between substitution rate and allelic polymorphism when the confounding effects of species and lineages are factored out.

At the cost of reducing statistical power, all analyses were repeated by excluding lineages belonging to a 'W' workshop number, as non-orthologous HLA lineages may involve different loci/lineages. In addition, we performed analyses to fully remove the potentially confounding effect of phylogenetic inertia, as different species and lineages may be differently related to each other due to shared evolutionary history. To eliminate the problems caused by the phylogenetic relatedness of allelic lineages, we focused exclusively on the variation within a single lineage and tested for correlations at the interspecific level. The phylogenetic relatedness of species was handled by appropriate comparative approaches that take into account the evolutionary trees of species [79]. We have chosen the most characterised lineage, DRB1*03, to demonstrate how the observed patterns can shape MHC polymorphism across species. To achieve the control for species-specific effects due to common descent in the interspecific context of DRB1*03, we applied the general method of phylogenetic analysis based on generalised least-squares (PGLS) models [80,81] that incorporated primate phylogeny [42] and statistical weights. In this PGLS exercise, we combined variance factors due to phylogenetic and weight effects as error terms in a form of a matrix exercise using the Q = V + *cW *equation, where V is the phylogeny matrix, W is the diagonal matrix of 1/weights and *c *is constant [80]. By varying *c *constant, we calculated the maximum likelihood of different combinations of the phylogeny and weight matrices. At the combination, which offered the highest fit to the data as revealed by the highest maximum likelihood, we determined the effect size of interest in the form of Pearson's r. This result was therefore obtained at the simultaneous adjustment for phylogenetic inertia and heterogeneous data quality, just as much as the data required [see [82]].

To avoid the potential problem arising from the combination of data across populations, we restricted our analyses to clear population-level studies. To achieve this, we repeated our procedures by strictly focusing on data from well-defined populations only. This criterion was met in 4 studies [[49,57,65], N. G. de Groot and Ronald E. unpublished data for a *Pan troglodytes *population from Sierra Leone], where information on the exact origin of individuals screened was available, and from this information we could be ascertain that the data correspond to a single population. Note that this restriction caused considerable reduction in sample size and statistical power. Therefore, for comparisons, we focus on effect sizes and not strictly on significance levels [83,84]. Moreover, the low sample size did not allow the construction of very complex models, thus it was not possible to simultaneously control for species- and lineage-specific effects. We, therefore, only included species-specific effects in the GLM design. However, we could use statistical weights as above.

We have chosen *DRB6 *sequences for the contrast analysis of non-functioning lineages, because previous studies showed that these lineages generally remain unexpressed, even if the alleles included do not have insertions, deletions or stop codons [85,86]. Accordingly, we have repeated the analyses with the *DRB6 *sequences that had been excluded from the above tests relying on functioning *DRB *lineages.

We avoided using dN:dS ratios in the statistical analyses. Correlations with ratios may be difficult to interpret because a given pattern may arise from the effect of the numerator, the denominator or the combination of the two [87]. Furthermore, our predictions were specifically applied to the independent roles of non-synonymous and synonymous substitution rates.

## Authors' contributions

LZG conceived of the study, participated in its design and coordination, collected data, performed the statistical analysis and drafted the manuscript. NGG collected data and drafted the manuscript. REB participated in the design of the study and drafted the manuscript. All authors read and approved the final manuscript.

## Supplementary Material

Additional file 1 **Primate DRB.** Click here for file

Additional file 2 **Refrences used in this study.** Click here for file
